# An Unusual Cause of Pancreatic Tumor

**DOI:** 10.7759/cureus.56421

**Published:** 2024-03-18

**Authors:** Mustafa A Sabri, Suhaila A Ahmed, Adiba Rakhange

**Affiliations:** 1 Gastroenterology, Rashid Hospital, Dubai, ARE; 2 Gastroenterology, Dubai Medical College for Girls, Dubai, ARE

**Keywords:** anti-tuberculosis medications, mycobacterium tuberculosis, fna, endosonography, pancreatic tuberculosis

## Abstract

Pancreatic tuberculosis (TB) is a rare condition that can be challenging to distinguish from other pancreatic neoplasms. We present the case of a 48-year-old Indian male who complained of persistent vague epigastric pain for two months. Other physicians saw him and treated him like a dyspeptic case. He gave a history of daily alcohol consumption.

Routine investigations, including amylase and lipase levels, were within normal limits. However, an abdominal ultrasound revealed a cystic lesion in the body of the pancreas, prompting further investigation. A subsequent abdominal CT scan revealed a tumor of 6x4 cm in diameter with solid and cystic components in the pancreatic body. Although tumor markers were not remarkable, inflammatory markers showed elevated levels of ESR (erythrocyte sedimentation rate) of 95 mm/hr and CRP (C-reactive protein) of 83 mg/L, with normal hemoglobin. Endosonography (EUS) with fine needle aspiration (FNA) was performed to achieve a definitive diagnosis. EUS was performed with a linear echoendoscope, which revealed the mass, which had solid and cystic components. Fluid was aspirated from the cystic part and FNA passes were performed in the solid part.

Microscopic examination and aspirated fluid culture confirmed the presence of *Mycobacterium tuberculosis*, while the solid part revealed caseation-indicated granulomas, indicative of TB.

The patient was promptly initiated on a seven-month course of three anti-TB medications, leading to normalization of ESR and CRP levels during the treatment period. A follow-up abdominal CT scan showed complete resolution of the pancreatic lesion, indicating successful management.

This case is rare and all the data in the literature is mainly in the form of case reports. Using EUS with FNA has transformed the diagnosis of pancreatic malignancy into a curable disease that could be easily managed with anti-TB medications.

## Introduction

Tuberculosis (TB), caused by *Mycobacterium tuberculosis*, continues to be the leading infectious disease, responsible for nearly 4,000 deaths per day worldwide. Although most TB cases are pulmonary, about 20% are extrapulmonary, with abdominal TB accounting for 10% of cases [1].

Abdominal TB can affect various intra-abdominal organs, and pancreatic involvement in TB is relatively rare. It occurs primarily during the multiorgan abdominal spread of the infection. The diagnosis of isolated pancreatic TB is often not suspected and can be misdiagnosed as pancreatic carcinoma, and the correct diagnosis is often made only after histological examination after resection [2].

The increase in reported cases of pancreatic TB in recent years can be attributed to the increased accessibility to advanced imaging tools and the development of various techniques to facilitate specimen collection from the pancreas [3]. Pancreatic tumors have various appearances and clinical presentations, often requiring the consideration of malignancy as a primary differential diagnosis.

However, with the advent of endosonography (EUS) and fine needle aspiration (FNA), the diagnostic process and tumor evaluation have improved significantly, improving sensitivity and specificity. This has led to an increase in reported cases of pancreatic TB in recent years. In certain cases, this advanced approach has the potential to overturn the initial provisional diagnosis of malignancy, favoring a more benign condition.

Despite these advances, awareness of pancreatic TB remains relatively low among physicians, leading to unsatisfactory treatment results and increased healthcare costs.

This case report presents a rare diagnosis in which a classical presentation of a pancreatic tumor was submerged under EUS and FNA, revealing an unexpected and favorable benign outcome. Consent from the patient was obtained for the collection and publication of relevant clinical data. All personal identifiers have been removed or appropriately anonymized to protect the privacy of the individuals involved.

## Case presentation

We present the case of a 48-year-old Indian gentleman who sought medical attention in May 2013 at Rashid Hospital due to intermittent vague epigastric pain radiating to the spine, not related to food, for two months. Initial evaluations by other physicians led to a provisional diagnosis of dyspepsia. He had a history of daily alcohol consumption. The patient exhibited moderate tenderness in the epigastric region on physical examination and was afebrile with stable vital signs.

Laboratory findings

A comprehensive set of initial investigations revealed normal hemoglobin levels (15.4 g/dL), normal white blood cell count (8,900 cells/μL), and normal platelets count (467,000 cells/μL), but a significantly elevated erythrocyte sedimentation rate (ESR) of 95 mm/hr and elevated C-reactive protein (CRP) levels at 83 mg/L. Liver function tests displayed elevated aspartate aminotransferase (AST) at 38 U/L, gamma-glutamyl transferase (GGT) at 351 U/L, and alkaline phosphatase (ALP) at 188 U/L. Additionally, alanine aminotransferase (ALT) was slightly elevated at 56 U/L, and bilirubin levels were within normal limits (1.1 mg/dL). Lipase levels were normal (80.80 U/L). Coagulation parameters were unremarkable.

Imaging

**Figure 1:**
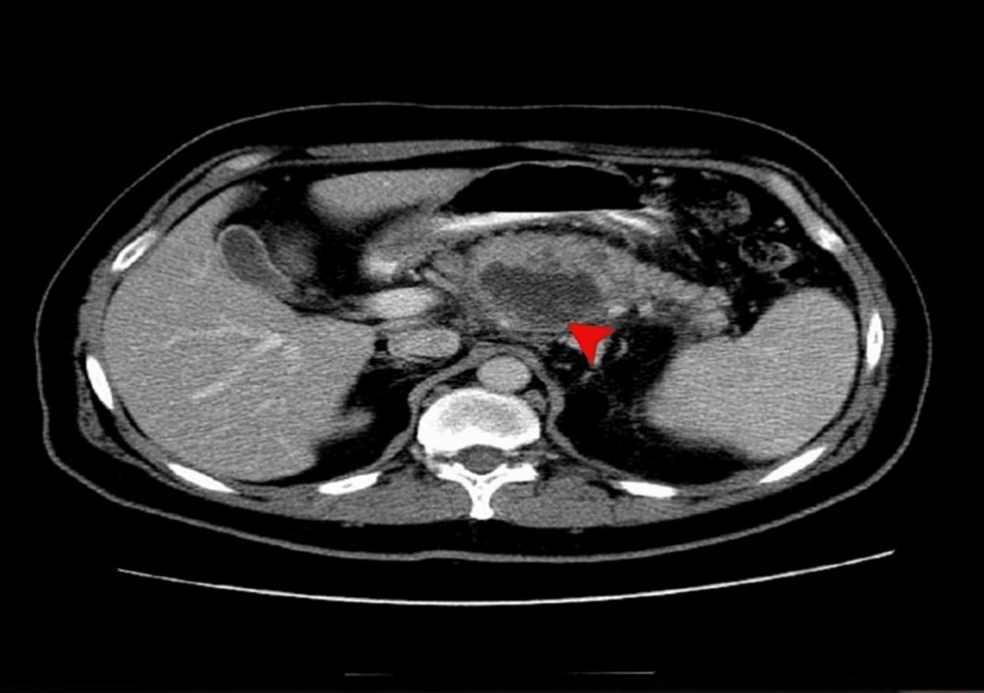
CT abdomen and pelvis with contrast (axial view) The red arrow represents a mixed density heterogeneously enhancing mass with large areas of necrosis in the body of the pancreas with encasement of the splenic artery and vein likely representing neoplastic pancreatic body mass - mucinous cystadenocarcinoma.

**Figure 2:**
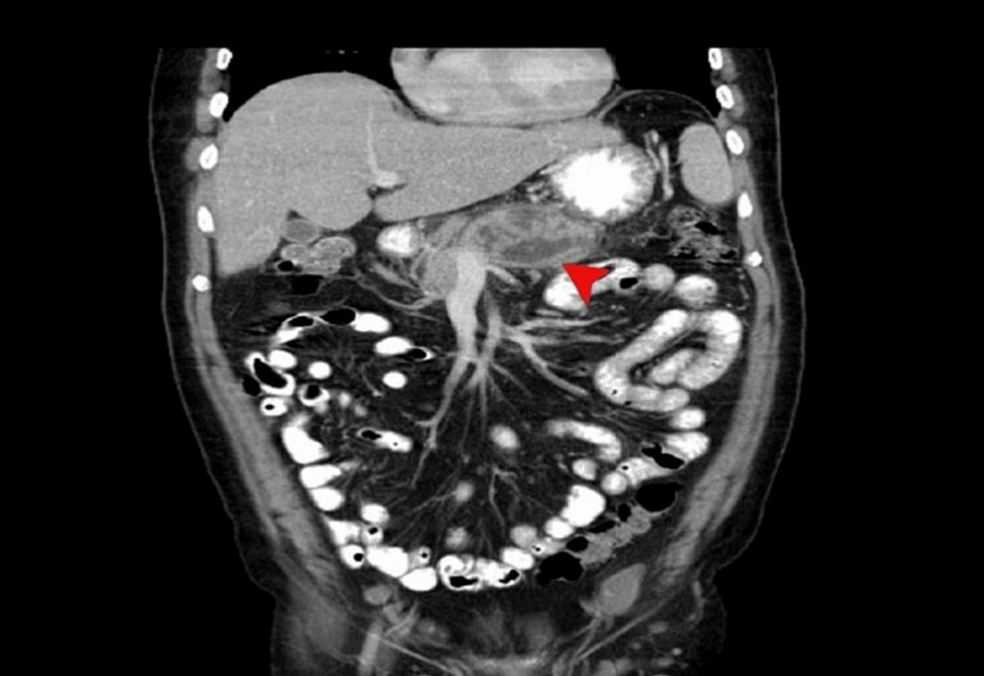
CT abdomen and pelvis with contrast (coronal view) The red arrow represents a mixed density heterogeneously enhancing mass with large areas of necrosis in the body of the pancreas with encasement of the splenic artery and vein likely representing neoplastic pancreatic body mass - mucinous cystadenocarcinoma.

A CT abdomen and pelvis with contrast was done; the results of which are shown in Figures 1-2.

**Figure 3:**
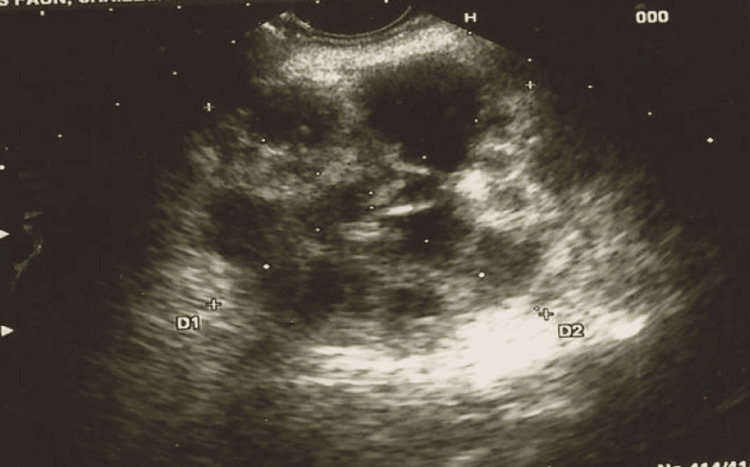
Endosonography Endosonography demonstrated the presence of a cystic mass (marked by the + sign), in the body of the pancreas, providing crucial information for further investigation.

An ultrasound examination of the abdomen was done as shown in Figure 3.

FNA

**Figure 4:**
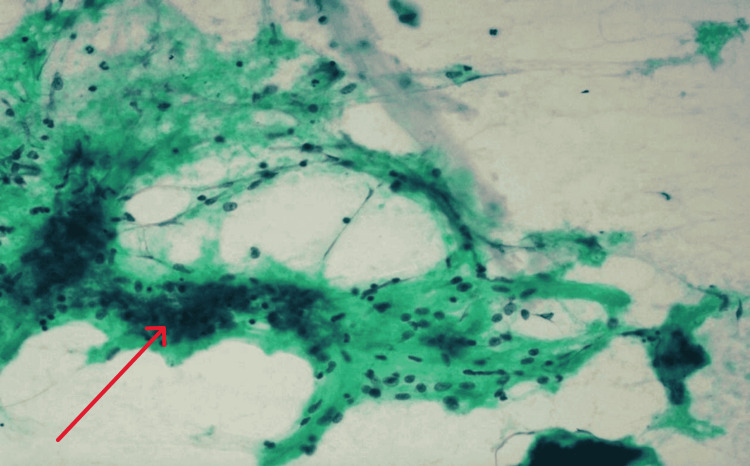
Results of cystic component aspiration Microscopic examination of the cyst fluid revealed the presence of inflammatory cells, comprising neutrophils, lymphocytes, and macrophages (red arrow) and occasional small yellowish-green bile pigments admixed with hemorrhage. Notably, no epithelial cells or malignant cells were observed.

The obtained samples from the FNA displayed moderate cellularity. Few acinar cells were identified with mildly enlarged nuclei. The background consisted of a diverse array of inflammatory cells, including neutrophils, macrophages, lymphocytes, and plasma cells. Furthermore, aggregates and scattered epithelioid histiocytes with necrotic material were observed, while no malignant cells were detected as shown in Figure 4.

Cyst Fluid Smear

Pancreatic cyst fluid was aspirated and sent for cytology, the results of which are shown in Figure 4.

Cyst Fluid Analysis

Amylase level in the cyst fluid was markedly elevated at 13,183 U/L, consistent with pancreatic origin.

Acid-Fast Bacilli (AFB) Staining

**Figure 5:**
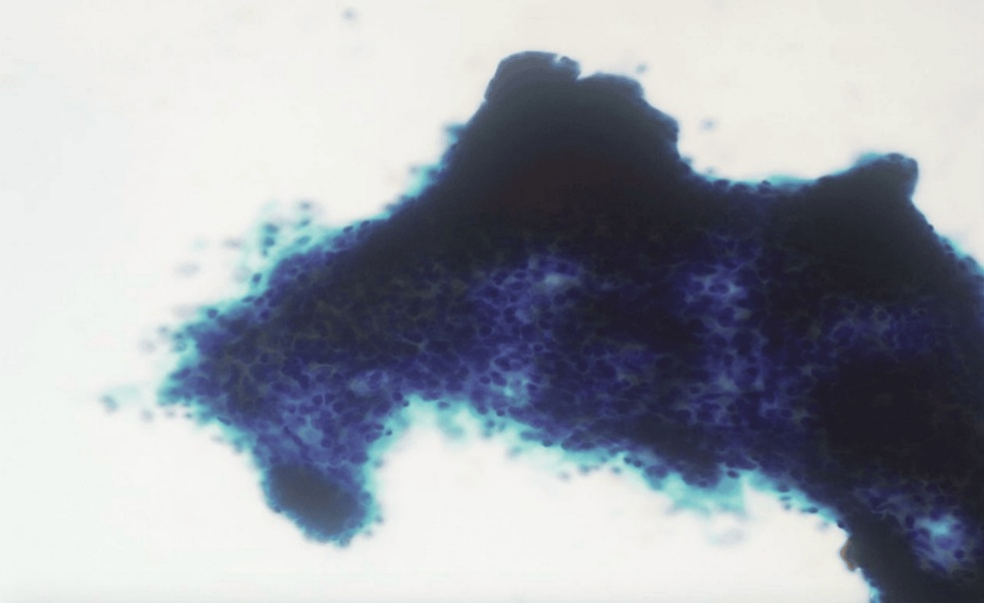
Acid-fast bacilli (AFB) staining The Ziehl-Neelsen stain for AFB showed a positive result in the cyst fluid, indicating the presence of AFB.

The results of AFB staining are shown in Figure 5.

Fluid Culture

The predominant pathogen identified in the fluid culture was *M. tuberculosis*. Additionally, scanty growth of other microorganisms, namely *Pseudomonas *and *Serratia*, was also noted.

These findings indicate a significant inflammatory response in the cystic component, along with the presence of *M. tuberculosis* and other microorganisms. The absence of malignant cells in the FNA provides reassurance regarding the non-malignant nature of the lesion.

The patient was diagnosed with pancreatic TB and was promptly commenced on a comprehensive anti-TB regimen, which included a seven-month course of the following medications: isoniazid, rifampicin, pyrazinamide, and pyridoxine (vitamin B6).

**Figure 6:**
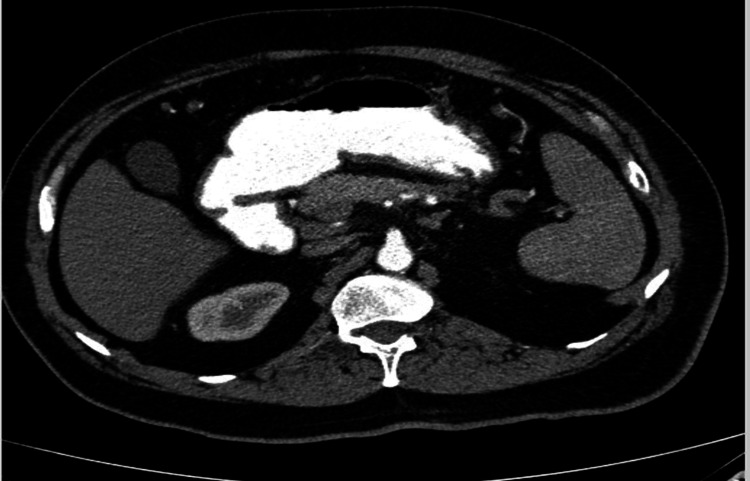
CT abdomen after treatment (axial view) The CT showed complete resolution of pancreatic lesions.

**Figure 7:**
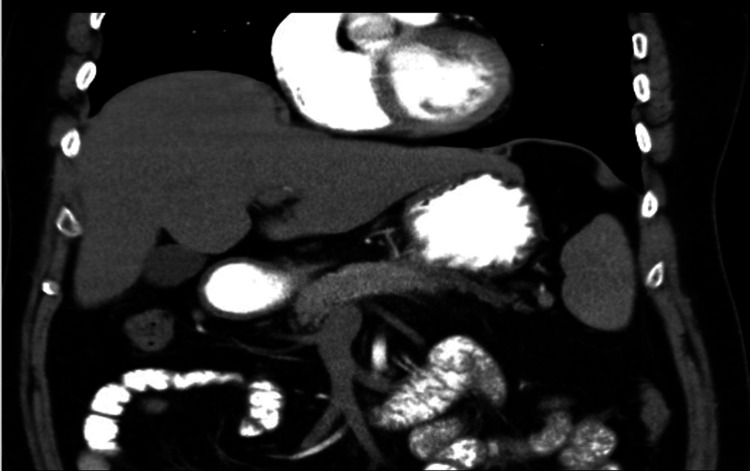
CT abdomen after treatment (coronal view) The CT showed complete resolution of pancreatic lesions.

After completing the prescribed treatment, the patient demonstrated a remarkable improvement in inflammatory markers. The initial ESR level, which was 95 mm/hr, significantly decreased to 20 mm/hr. Previously elevated CRP levels, measuring 83 mg/L, returned to the normal range. This significant decrease in both ESR and CRP levels indicated a successful response to the anti-TB treatment. Following the completion of the treatment course, the following CT was obtained as seen in Figures 6-7.

## Discussion

TB has resurfaced as a significant concern over the past two decades, largely attributed to the increasing prevalence of immunodeficiency and immunosuppression. Factors such as globalization, evolutionary changes in bacterial biology, and the development of drug resistance have further contributed to its resurgence [1,4].

Among extrapulmonary sites, TB affecting the gastrointestinal tract is the sixth most common, with abdominal TB accounting for approximately 10% of cases [5,6]. Isolated pancreatic TB is very rare, even in endemic areas. From 1966 to 2004, a total of 116 cases of pancreatic TB were identified through a comprehensive search of the MEDLINE database. Subsequently, from 2005 until the present year, an additional 49 cases have been reported.

The presence of a high concentration of pancreatic enzymes could be a protective mechanism against invasion by mycobacteria [7]. Immunosuppressive states, such as diabetes and medications, are considered potential risk factors for isolated pancreatic TB. The connection between alcohol abuse, chronic pancreatitis, and pancreatic TB has not yet been fully elucidated and requires further investigation.

The possible mechanisms of the spread of *M. tuberculosis* to the pancreas are lymphohematogenous spread from occult lung lesions or direct spread from adjacent organs [8,9]. The pathological appearance of the pancreas in most cases is suggestive of a pancreatic tumor. The head and uncinate processes of the pancreas are the most frequent sites for pancreatic TB and pancreatic cancer, which presents a diagnostic challenge.

Clinically, isolated pancreatic TB often presents with symptoms such as epigastric pain, fever, dyspepsia, weight loss, weakness, and night sweats, which mimic pancreatic cancer.

Laboratory investigations typically reveal elevated levels of ESR and CRP. Cholestatic features may manifest if the bile duct is involved. The best diagnostic tools for the diagnosis of pancreatic TB are imaging; ultrasound, contrast CT scan followed by EUS and FNA of the pancreatic lesion and or any other lymph nodes, for AFB microscopy and culture, cytology for malignant cells, inflammatory cells, granuloma [10]. Imaging without tissue diagnosis is not sufficient to differentiate between pancreatic TB and malignant pancreatic tumor.

Previously, obtaining a tissue sample for diagnosis involved invasive procedures such as CT-guided biopsy or laparoscopic biopsy, with a higher risk of complications. However, the advent of EUS-guided FNA has revolutionized the diagnostic approach, offering a less invasive and more accurate means of obtaining tissue samples [11,12].

Standard treatment for pancreatic TB involves a regimen of anti-TB medications for 6-12 months, with regular follow-up through imaging and laboratory investigations to assess response to treatment and tumor resolution [8]. This approach has been proven to be effective in managing the disease and minimizing complications.

## Conclusions

Pancreatic TB, though rare, remains an important diagnostic challenge. A high index of suspicion is vital for timely diagnosis and appropriate management. The incorporation of advanced imaging and EUS-guided FNA has markedly enhanced diagnostic precision while minimizing invasiveness. Ensuring prompt and appropriate treatment is essential for achieving positive outcomes and the overall well-being of the patient.
